# Supplementary material for: Scale-Up of Doppler to Improve Intrapartum Fetal Heart Rate Monitoring in Tanzania: A Qualitative Assessment of National and Regional/District Level Implementation Factors
Source: Int J Environ Res Public Health. 2020 Mar 16;17(6):1931. doi: 10.3390/ijerph17061931 (PMC7142453; doi:10.3390/ijerph17061931)
Supplement: Supplementary file 1 [file ijerph-17-01931-s001.pdf]

Doppler for Improved Intrapartum Care in Tanzania

Key Informant Interview Questionnaire

Introduction: This study looks at potential barriers and facilitators to integration of Doppler into intrapartum care in the public health sector in Tanzania. The primary research question leading the questionnaire is:

- What are the barriers and facilitators to use of / scale up of the Doppler to improve facility-based intrapartum care in Tanzania?

This interview tool will capture perspectives of key informants using a qualitative, in-depth interview format.

Directions: Introduce yourself. Administer informed consent, sign the form and interview the key informant. Attach the informed consent to this form.

Introduction: Hello, I am ________. I would like to talk to you about your views on the potential facilitators and barriers to integrating the Doppler into intrapartum care in government health facilities which offer maternity services in Tanzania. Your answers will be collated with other key stakeholders' views to inform a qualitative analysis of the potential facilitators and barriers to scale up of the Doppler for intrapartum services in Tanzania.

Date of Interview ____

Name of Key Informant ____

Position / Job Title ____

Institutional Affiliation ____

Note start time of interview:

Section 1. Background characteristics

1. What is your current job title?
2. How long have you been working for ______________________ ?

Section 2. Affordability ____________________________________

1. Can you tell me, what do you feel is the current resource availability for improving intrapartum care in the government health facilities in Tanzania? At national level? At district level? At facility level?

Probes: *where do financial resources for improving intrapartum care in Tanzania come from? Are they cyclical? What would be the next opportunity to find funds to improve intrapartum care?*

2. I want to use another example of improving intrapartum and newborn care in Tanzania to compare to that of using Doppler for improved intrapartum care. Do you have any experience with rollout of the HBB program in Tanzania? In your understanding, what financial and other resources were needed from external donors for rollout of HBB initiative? From MOHCDGEC?

Probes: *How do you think the resources needed for scaling up Doppler for intrapartum care might be similar to HBB? How might it be different?*

3. We have discussed the resources needed and the example of how HBB was scaled up in Tanzania. What affordability barriers do you anticipate if Doppler were to be scaled up to all facilities providing maternity services?

Probes: *How could those financial barriers be strategically anticipated? What would be some ways to work around them?*

4. In addition to barriers, there might be facilitators which would help move the initiative to integrate Doppler into intrapartum care. What facilitators for financing this initiative do you anticipate?

Probes: *How could those financial facilitators be strategically anticipated? What would be some ways to enhance them?*

Section 3: Practicability ____

1. In your opinion, what are current priorities for improving intrapartum care in public health facilities in Tanzania?

Probes: *What are the ones detailed in policy/ Roadmap? What are some of the more politically driven agenda areas which might or might not be in the Roadmap? Where would improving intrapartum care fall in terms of national priorities? Regional or district priorities?*

2. We have discussed financial resources which might be required to scale up Doppler for intrapartum care. What other, non-financial resources would be needed to scale up use of Doppler in intrapartum care in Tanzania? From MOHCDGEC? From NGO's? From external donors?

3. What human resource and systems barriers do you anticipate if Doppler were to be scaled up to all facilities providing maternity services?

Probes: *Do you think the biggest barrier would be human resources? Health care provider adoption? Finances? Quality of usage?*

4. What facilitators do you anticipate?

Probes: What d*o you think the biggest facilitator to adopting Doppler for improved intrapartum care? Do you think professional associations have a role to play? Community?*

Section 4. Acceptability ____

1. How do you feel that use of Doppler in intrapartum care aligns with current national priorities for maternal and newborn care?

Probe: Are there competing priorities? Are there synergistic factors?

2. Is it likely that using Doppler for intrapartum care will be acceptable to the District medical authorities? facility managers? nurse/midwives providing intrapartum care?

Probe: *Why or why not?*

Note end time of interview ________
